# Evaluation of the Postoperative Quality of Recovery Scale test and re-test in Swedish among healthy volunteers

**DOI:** 10.12688/f1000research.9740.1

**Published:** 2016-10-21

**Authors:** Pether Jildenstål, Johan Eriksson, Margareta Warren Stomberg, Jan G. Jakobsson

**Affiliations:** 1 Institute of Health and Care Sciences, The Sahlgrenska Academy, University of Gothenburg, Gothenburg, Sweden; 2 Department of Anaesthesiology, Surgery and Intensive Care, Sahlgrenska University Hospital, Örebro, Sweden; 3 Department of Anaesthesiology and Intensive Care, Örebro University Hospital, Örebro, Sweden; 4 Department of Anaesthesiology, Queen Silvia Children´s Hospital, Gothenburg, Sweden; 5 Institution for Clinical Science, Karolinska Institute,, Danderyds University Hospital, Stockholm, Sweden

**Keywords:** anaesthesia, recovery, quality of recovery, PostopQRS, volunteers, re-test

## Abstract

*Introduction *

Patient outcome measures are required to assess the quality of healthcare. Tools for a patients’ self-assessment of quality of recovery, during perioperative care, have been developed during the last decade. The Postoperative Quality of Recovery Scale (PostopQRS) questionnaire is one of the most well-accepted and validated tools available. Here we assess the PostopORS questionnaire in Swedish.

*Methods*

Sixty-one students from the Bachelor Program in Nursing, (50 female and 11 male; mean age, 25; range, 21-46) filled in the Swedish translation of the PostopQRS questionnaire twice. They also evaluated whether they found the queries easy to understand and respond to **. **

*Results*

The participants found the Swedish translation of the PostopQRS questionnaire easy to read and understand. There were minor differences in test responses between the initial test and the re-test 48 hours later. We found that the PostopQRS questionnaire has some background noise; 12 out of 61 participants (20%) reported mild pain, 25 (41%) scored some depression and 33 scored mild anxiety (54%). The cognitive domain showed a learning effect between tests in “word recall” and “word generation”, while “digit recall forward” and “digit recall backward” showed no change. We found a difference in cognitive test performance with age; younger participants had higher mean cognitive test scores compared to participants >30 years. Overall, nine participants showed a decrease in re-test scores; two experienced a mild increase in pain; one experienced a mild increase in anxiety; and six performed more poorly on cognitive tests.

*Conclusion*

The Swedish translation of the PostopQRS was found to be adequate for use in the assessment of quality of recovery, and the questions were well understood by participants. Our study shows the importance of baseline testing for assessment of recovery, since recovery is assessed as a return to or improvement in each individual’s baseline score.

## Introduction

There is an increasing interest in identifying patient outcome measures ^1, 2^ to best assess the quality of patient recovery ^3^. Several tools have been developed to achieve this goal ^4^. The Postoperative Quality of Recovery scale (PostopQRS) questionnaire was developed in 2010 and has been widely accepted as an effective tool for the self-assessment of patients’ quality of recovery ^3, 5^. The PostopQRS homepage provides detailed information around how to use the test and available languages ( http://www.postopqrs.com/). The PostopQRS assesses patient recovery compared to a unique individual preoperative, baseline score. This is somewhat in contrast to other tests, e.g. the Quality of Recovery Scale, where the absolute score is commonly used to describe recovery. Moreover, Myles *et al*. recently published recommendations around the minimal clinically important difference ^4^. The PostopQRS questionnaire addresses multiple domains, including nociception, emotion, day-to-day activities, cognition and satisfaction. The cognitive domain consists of a five tests, as follows: orientation, digit recall forwards, digit recall backwards, letter forwards and word generation. These tests have been shown to be effective in assessing cognitive performance ^6^. There has been a discussion around how individual cognitive re-test scores should be evaluated, and an amended technique, including a tolerance factor of -1 to -3, is now recommended ^7, 8^. The PostopQRS is an attractive tool for assessing the quality of recovery following general, as well as local, anaesthesia ^9^.

The aim of the present study was to validate the Swedish translation of the PostopQRS in a test and re-test study in healthy volunteers.

## Methods

Ethical approval was obtained from the Ethics Committee of Stockholm (January 20 ^th^ 2016; approval no., Dnr 20152015/2163-31/4, Sweden) prior to the start of the study. Oral informed consent was obtained from 65 adult students at the University of Gothenburg, Sahlgrenska Academy, aged between 21 and 39 years. The study was conducted between 29 ^th^ August 2016 and 2 ^nd^ September 2016.

Exclusion criteria included the inability to complete the questionnaire, due to mental disabilities, hearing impairment, any form of substance abuse or not having Swedish as their native language.

Four anaesthetic nurses and senior lecturers trained in interview techniques performed the tests.

The PostopQRS tests (Data availability; http://www.postopqrs.com/) were performed on two occasions. Tests were initially undertaken face-to-face on the day of inclusion to establish a baseline, and then on day 2, 48 hours after the initial baseline test. The second test was performed by telephone.

The tests were all performed in a quiet environment, free from distraction. The participants completed all sections of the PostopQRS on both occasions. The questions and answers were read from the prescribed PostopQRS script. During the initial test, all participants also read the question by themselves, with the exception of the cognitive tests.

All participants were explicitly asked whether they had any hesitation regarding the understanding of the questions, both when provided verbally and by reading. The question about understanding the queries was asked on both occasions, although during the telephone re-test only verbal evaluation was possible.

All questions were translated to Swedish, The letters used for the word generation test were D and S was used; D for the initial base-line test and S for the 48-hour re-test.

The physiological domain, which assesses vital signs, were not included in this study.

### Statistical analysis

Data were collected from the interview personnel before submission to the data administrator for analysis. Descriptive statistics in the demographics section is expressed as numbers, mean±SD and range; minimum to maximum values. Participants were divided into three different groups depending on age (20–24 years, 25–29 years, and >30 years). Significance testing was performed using Wilcoxon signed-rank test or Kruskal-Wallis test where appropriate, and were analysed using SPSS version 23.0 for Windows (SPSS Inc, Chicago, IL, USA). A two-tailed P value >0.05 was considered significant. Results are presented for the entire cohort and the three age groups.

## Results

Raw data from the test and retest Click here for additional data file. Copyright: © 2016 Jildenstål P et al. 2016 Data associated with the article are available under the terms of the Creative Commons Zero "No rights reserved" data waiver (CC0 1.0 Public domain dedication).

Swedish translation of the PostopQRS questionnaire Click here for additional data file. Copyright: © 2016 Jildenstål P et al. 2016 Data associated with the article are available under the terms of the Creative Commons Zero "No rights reserved" data waiver (CC0 1.0 Public domain dedication).

We included 65 students in the study. The initial test was performed face-to-face and the retest was by phone interview. Four (n=4) students could not reached by phone for the re-test; thus only 61 subjects were included in the result analysis. The mean age for the cohort was 25 years and the majority of the participants were female ( Table 1).

**Table 1.** Demographics for the participants of the PostopQRS-test validation.

Characteristics	Participants *n=*61
Sex, F/M *(n)*	50/11
Age, years, mean ± SD	25.31 ± 5.08
Age, years, min-max	21–46
Age, years, 20–24 *(n)*	32
Age, years, 25–29 *(n)*	22
Age, years, 30+ *(n)*	7

All participants explicitly expressed that the questions were easy to read and understand, and also easy to understand when asked orally.

The overall results of the test and re-test results for the different domains [nociceptive, emotion, day-to-day activities (ADL domain), and cognition] are presented in Table 2.

**Table 2.** Primary results from the test and the re-test 48 hours later.

	Mean	SD	Median	Min. Max.	Increase No. Subj.	Decrease No. Subj.
***Nociceptive domain***					*2 subjects with* *failure*	
Pain BL	1.2	.5	1	1–3	2	4
PONV BL	1		1			
***Emotional domain***					*1 subject with* *failure*	
Depression	1.4	.5	1	1–3		4
Anxiety	1.6	.6	1	1–3	1	16
***Cognition domain***						*6 subjects with* *failure*
Orientation						
Digit recall forwards	4.7	1	5	3–6	24	2*
Digit recall backwards	3.7	1	3	1–6	7	3*
Word recall	7.2	1.8	7	4–13	40	1*
Word generation	8.1	2	8	2–14	53	0
***ADL domain***						
Stand	3		3			
Walk	3		3			
Eat	3		3			
Dress	3		3			
***Overall perception***						
Ability to work compared to before your surgery?	1		1			
Ability to undertake daily living activities	1	.1	1	1–2		
Clarity of thought now compared to before your surgery?	1		1			
Satisfied with the anaesthetic	1		1			

*Taking the correction factor suggested by Royse *et al.,* 2013.

In the initial test (base-line), 12 out of 61 (20%) participants reported mild pain, 25 (41%) scored mild depression and 33 (54%) scored mild anxiety.

All participants in the study scored the maximum score in orientation, at base-line and at the 48-hour re-test. Digit recall forward, digit recall backwards, word recall and word generation had a median value score of 5, 3, 7 and 8, respectively, at base-line, with ranges 3, 5, 9 and 12 (see Table 2). Word recall and word generation showed both a significant improvement in the re-test ( Table 3), which may be characterised as a ‘learning effect’. When cognitive test performance was separated by age, a numeric difference was seen in overall performance ( Table 4– Table 6); the absolute scores decreased with age, and the learning effect diminished ( Table 7, Table 8).

**Table 3.** Cognitive scores in participants, all ages.

	Baseline, mean ± SD (median, range)	T _48_, mean ± SD (median, range)	Change, mean ± SD (median, range)	Wilcoxon *P*
K1 Orientation	3 ± 0 (3, -)	3 ± 0 (3, -)	0 ± 0 (3, -)	-
K2 Digit recall forwards	4.72 ± 0.99 (5, 3)	4.87 ± 1.01 (5, 3)	0.15 ± 1.22 (0, 6)	0.30
K3 Digit recall backwards	3.69 ± 1.01 (3, 5)	3.48 ± 1.03 (3, 4)	-0.21 ± 0.84 (0, 4)	0.07
K4 Word recall	7.23 ± 1.78 (7, 9)	8.39 ± 1.55 (8, 7)	1.16 ± 1.61 (1, 8)	<0.01
K5 Word generation	8.13 ± 2.06 (8, 12)	11.49 ± 2.53 (12, 11)	3.36 ± 2.43 (4, 11)	<0.01

**Table 4.** Cognitive scores in participants, age 20–24 years.

	Baseline, mean ± SD (median, range)	T _48_, mean ± SD (median, range)	Change, mean ± SD (median, range)	Wilcoxon *P*
K1 Orientation	3 ± 0 (3, -)	3 ± 0 (3, -)	0 ± 0 (3, -)	-
K2 Digit recall forwards	4.91 ± 0.86 (5, 3)	4.97 ± 0.93 (5, 3)	0.06 ± 1.1 (0, 5)	0.64
K3 Digit recall backwards	3.94 ± 1.01 (4, 4)	3.59 ± 1.10 (3, 4)	-0.34 ± 0.94 (0, 4)	0.07
K4 Word recall	7.59 ± 1.78 (7.5, 8)	8.91 ± 1.61 (9, 6)	1.31 ± 1.53 (1, 6)	<0.01
K5 Word generation	8.59 ± 2.18 (8, 10)	11.97 ± 2.46 (12, 11)	3.38 ± 2.43 (3.5, 10)	<0.01

**Table 5.** Cognitive scores in participants, age 25–29 years.

	Baseline, mean ± SD (median, range)	T _48_, mean ± SD (median, range)	**Change**, mean ± SD (median, range)	Wilcoxon *P*
K1 Orientation	3 ± 0 (3, -)	3 ± 0 (3, -)	0 ± 0 (3, -)	-
K2 Digit recall forwards	4.55 ± 1.14 (4, 3)	4.59 ± 1.18 (5, 3)	0.05 ± 1.43 (0, 6)	0.88
K3 Digit recall backwards	3.55 ± 1.06 (3, 4)	3.46 ± 1.01 (3, 3)	-0.09 ± 0.75 (0, 3)	0.56
K4 Word recall	6.96 ± 1.91 (7, 9)	8.09 ± 1.15 (8, 5)	1.14 ± 1.91 (1, 8)	0.02
K5 Word generation	8.00 ± 1.35 (8, 5)	11.32 ± 2.57 (11, 11)	3.32 ± 2.46 (3.5, 9)	<0.01

**Table 6.** Cognitive scores in participants, age 30+ years.

	Baseline, mean ± SD (median, range)	T _48_, mean ± SD (median, range)	**Change**, mean ± SD (median, range)	Wilcoxon *P*
K1 Orientation	3 ± 0 (3, -)	3 ± 0 (3, -)	0 ± 0 (3, -)	-
K2 Digit recall forwards	4.43 ± 0.98 (4, 3)	5.29 ± 0.49 (5, 1)	0.86 ± 0.90 (1, 2)	0.06
K3 Digit recall backwards	3.00 ± 0.00 (3, 0)	3.00 ± 0.58 (3, 2)	0.00 ± 0.58 (0, 2)	-
K4 Word recall	6.43 ± 0.98 (6, 3)	7.00 ± 1.41 (7, 4)	0.57 ± 0.79 (0, 2)	0.10
K5 Word generation	6.43 ± 2.64 (7, 7)	9.86 ± 2.34 (9, 6)	3.43 ± 2.70 (4, 8)	0.03

**Table 7.** Comparison of age-groups at baseline in the cognitive domain.

	Age 20–24, median (range)	Age 25–29, median (range)	Age30+, median (range)	Chi-square* *P*
K1 Orientation	3, -	3, -	3, -	-
K2 Digit recall forwards	5 (3)	4 (3)	4 (3)	0.27
K3 Digit recall backwards	4 (4)	3 (4)	3 (0)	0.03
K4 Word recall	7.5 (8)	7 (9)	6 (3)	0.12
K5 Word generation	8 (10)	8 (5)	7 (7)	0.15

*Kruskal-Wallis Test

**Table 8.** Comparison of age-groups at T _48_ in the cognitive domain.

	Age 20–24, median (range)	Age 25–29, median (range)	Age30+, median (range)	Chi-square* *P*
K1 Orientation	3, -	3, -	3, -	-
K2 Digit recall forwards	5 (3)	5 (3)	5 (1)	0.34
K3 Digit recall backwards	3 (4)	3 (3)	3 (2)	0.42
K4 Word recall	9 (6)	8 (5)	7 (4)	0.01
K5 Word generation	12 (11)	11 (11)	9 (6)	<0.01

*Kruskal-Wallis Test

All participants, except one, scored full capacity in day-to-day activates and overall perception on both test occasions.

A decrease in pain and depression was seen in four participants, and 16 participants scored lower in anxiety at in the re-test ( Table 2). A learning effect was seen in word recall and word generation. There were overall nine participants (15%) that showed a decrease in test scores; two participants experienced a mild pain increase, one experienced a mild increase in anxiety, and six demonstrated a poorer performance on cognitive tests ( Table 2).

## Discussion

We observed that the Swedish translation of the PostopQRS is easy to read and understand. The test and the re-test 48 hours later showed minor alterations. There was background noise and a learning effect in word recall and generation tests between the two test occasions. The cognitive tests showed huge individual variability in scores emphasising the importance of baseline testing, since PostopQRS defines recovery as whether an individual has regained their base-line score. The PostopQRS does not assess an absolute value; it defines recovered/not recovered as a return to base-line performance or better test score.

The PostopQRS was developed by an international team in 2010, and since then has been validated and is now considered a robust test tool ^3, 5, 10^. The questionnaire has been translated into several languages ( http://www.postopqrs.com/). The Japanese translation was studied in patients by Naito *et al*. ^11^, who considered it a feasible tool for assessing recovery after surgery, despite having some limitation in ceiling effect in a high number of questions. The questionnaire was also recently translated into Chinese by Bu *et al*. ^12^, who concluded that the translated PostopQRS tool was robust, but showed that the Quality of Recovery scale ^13^ had a higher validity and was faster to perform.

The importance of a baseline test was also shown by Lindqvist *et al*. ^14^, who studied patients scheduled for breast cancer surgery and showed that the baseline test was significantly affected. In addition, several patients did not have a baseline score that was sufficient for the assessment of the recovery process. The learning effect was also seen in a volunteer study by Royse *et al*. ^7^.

The impact on age has also been addressed by Royse *et al*. The authors found only minor age effects on the recovery process; however, they did not investigate the explicit test results ^9^. Both age and time of day have been shown to influence more complex word tests, with older patients performing better in the morning in contrast to younger patients, who exhibit better results in the evening ^15^. In this study, we used the letters ‘D’ and ‘S’ for word generation. It has been suggested that the letters should be changed for the word generation test, and different words, letters and numbers should be chosen for the recall domain tests in re-tests, in order to reduce any learning effect. In agreement with Rosye *et al*., we used face-to-face interviews for the initial test and phone interviews for the re-test ^7^. Royse *et al*. did not find that phone interviews had a significant impact on the results.

There are several limitations of the present study. The participants in our study were young healthy volunteers; thus, the participants were not exposed to any form of intervention, such as surgery and anaesthesia. We made only one follow-up after 48 hours and did not perform re-tests at any additional time-points. It should also be acknowledged that we did not compare the PostopQRS questionnaire with any other assessment tool. We do consider the PostopQRS a well-established recovery assessment tool ^3, 13^, and also find that the Swedish version could work well in determining patients’ quality of recovery.

In summary, we observed that the Swedish translation of the PostopQRS is easy to read and understand, and preoperative baseline testing is of importance to define each individuals score profile. The test and re-test performed showed low variability, which was observed by the majority of participants performing equally on both tests. However, a learning effect in the word recall and word generation cognitive tests was observed. In conclusion, we believe that the PostopQRS is a feasible tool for assessment of recovery.

## Data availability

The data referenced by this article are under copyright with the following copyright statement: Copyright: © 2016 Jildenstål P et al.

Data associated with the article are available under the terms of the Creative Commons Zero "No rights reserved" data waiver (CC0 1.0 Public domain dedication). 

F1000Research: Dataset 1. Raw data from the test and retest, 10.5256/f1000research.9740.d139660 ^16^

F1000Research: Dataset 2. Swedish translation of the PostopQRS questionnaire, 10.5256/f1000research.9740.d139661 ^17^
