# Supplementary material for: Evaluation of the Postoperative Quality of Recovery Scale test and re-test in Swedish among healthy volunteers
Source: F1000Res. 2016 Oct 21;5:2549. [Version 1] doi: 10.12688/f1000research.9740.1 (PMC5310376; doi:10.12688/f1000research.9740.1)
Supplement: Swedish translation of the PostopQRS questionnaire [file f1000research-5-10501-s0001.tgz › 62aefcd0-724f-4d14-83c4-668ea23ae5b7_The_post-operative_quality_recovery_scale,_a_Swedish_translation__.doc]

**The post-operative quality recovery scale**, a Swedish translation

**I Fysiologi –**

P1. Patientens systoliska blodtryck. *Ange siffran som stämmer med hur du bedömer patienten*

1 = < 70 eller > 180

2 = 70 - 89 eller 141 - 180

3 = 90 - 140

P2. Patientens hjärt/pulsfrekvens. *Ange siffran som stämmer med hur du bedömer patienten*

1 = < 35 eller > 140

2 = 35 - 44 eller 101 - 139

3 = 45 - 100

P3. Patientens kroppstemperatur. *Ange siffran som stämmer med hur du bedömer patienten*

1 = < 35 eller > 39

2 = 35 - 35.9 eller 37.7 - 38.9

3 = 36 - 37.6

P4. Patientens andningsfrekvens. *Ange siffran som stämmer med hur du bedömer patienten*

1 = < 5 eller >30

2 = 5 - 9 eller 21 - 30

3 = 10 - 20

P5. Patientens SpO_2_ med eller utan syrgas. *Ange siffran som stämmer med hur du bedömer patienten*

1 = Episoder med SpO_2_ < 90 % trots syrgas

2 = Episoder med SpO_2_ < 95 % trots syrgas

3 = O_2_ syrgas enligt stående ordination eller ingen syrgas

P6. Hur patienten klarar sin luftväg. *Ange siffran som stämmer med hur du bedömer patienten*

1 = Patienten behöver tub, lma, svalgtub eller motsvarande

2 = Patienten behöver någon form av hjälp för att klara luftvägen

3 = Patienten upprätthåller själv fri luftväg

P.7 Agitation/oro. *Ange siffran som stämmer med hur du bedömer patienten*

1 = Patienten är påtagligt orolig/agiterad

2 = Patienten är av och till orolig/agiterad

3 = Patienten visar inga tecken på oro/agitation

P8. Medvetandegrad/vakenhet. *Ange siffran som stämmer med hur du bedömer patienten.*

1 = Patienten är ej väckbar

2 = Patienten är väckbar av ljud och fysiskt stimuli

3 = Patienten är fullt vaken

P9. Var vänlig och peka på din näsa och lyft huvudet. *Ange siffran som stämmer med hur du bedömer patienten*

1= Patienten Reagerar ej på uppmaning/följer ej kommando/inga försök till rörelse

2 = Patienten reagerar men slutför inte hela instruktionen

3 = Patienten följer/”lyder” instruktioner

II. **Nociception**

N1. Jag kommer att visa dig ett antal olika ansikten och vill att du anger vilket ansikte, siffra eller beskrivning som bäst beskriver din smärta just nu?

*Visa de olika ansiktena för patienten och anteckna den siffra som överensstämmer med patientens svar.*

1. Ingen smärta
2. Lätt smärta
3. Måttlig smärta
4. Svår smärta
5. Värsta tänkbara smärta

N2. Jag kommer att visa dig ett antal olika ansikten och vill att du anger vilket ansikte, siffra eller beskrivning som bäst beskriver ditt illamående eller kräkningar just nu.

*Visa de olika ansiktena för patienten och anteckna den siffra som överensstämmer med patientens svar.*

1. Inget illamående, inga kväljningar eller kräkningar
2. Lätt illamående, inga kväljningar eller kräkningar
3. Måttligt illamående och/eller kväljningar/kräkningar
4. Svårt illamående och/eller kväljningar/kräkningar
5. Kontinuerliga kväljningar/kräkningar
III. **Emotion**

E1. Jag kommer att visa dig ett antal olika ansikten och vill att du anger vilket ansikte, siffra eller beskrivning som bäst beskriver i vilken utsträckning du känner dig ledsen, nedstämd eller deprimerad just nu.

1. Inte alls deprimerad/ledsen
1. Lite deprimerad/ledsen
2. Något deprimerad/ledsen
3. Ganska deprimerad/ledsen
4. Extremt deprimerad/ledsen

E2. Jag kommer att visa dig ett antal olika ansikten och vill att du anger vilket ansikte, siffra eller beskrivning som bäst beskriver i vilken utsträckning du känner dig ängslig eller nervös just nu.

1. Inte alls ängslig/nervös
2. Lite ängslig/nervös
3. Något ängslig/nervös
4. Ganska ängslig/nervös
5. Extremt ängslig/nervös

**IV.**	Kognition

| **Kognitiva faktorer** |  |
| --- | --- |
| **K1. Berätta vad du heter, vilken stad vi befinner oss i och när du är född.**  **Anteckna antalet korrekta svar.**    Poäng_____________ |  |
| **K2. Jag kommer att läsa upp ett antal siffror för dig. Lyssna noggrant. När jag är färdig vill jag att du upprepar siffrorna i samma ordning som jag läste upp dem. Till exempel, om jag sa 1, 2, 3 ska du säga 1, 2, 3.**  Läs upp siffrorna med en hastighet en siffra/sekund. Avsluta uppgiften så fort patienten har svarat fel. Registrera poängen för den senaste helt korrekta återgiva raden.  1 6,7  2 2,5,3  3 6,3,8,2  4 5,7,3,6,1  5 4,3,9,5,2,8  6 1,7,9,5,3,2,4  POÄNG______ |  |
| **K3. Jag kommer att läsa upp ytterligare ett antal siffror för dig, men den här gången vill jag att du upprepar siffrorna i omvänd ordning. Till exempel, om jag sa 1, 2, 3 ska du säga 3, 2, 1.**    1 5,6  2 3,7,4  3 5,9,1,3  4 7,6,8,2,4  5 3,6,1,5,9,2  6 1,4,8,6,3,9,2  POÄNG______ |  |
| **K4. Jag kommer att läsa upp ett antal ord för dig. Lyssna noggrant. När jag är färdig vill jag att du upprepar så många av orden som du kommer ihåg. Du kan säga dem i vilken ordning du vill. Om du är osäker på om du redan har sagt ett ord, säg det igen för säkerhets skull.**  Läs upp orden med en hastighet av ett ord/sekund. Registrera antal korrekt återgivna ord.  **Trumma, gardin, klocka, kaffe, skola, förälder, måne, trädgård, hatt, bonde, näsa, kalkon, färg, hus, flod.**    POÄNG_______ |  |
| **K5. Jag kommer att säga en bokstav och jag vill att du på 30 sekunder anger så många ord du kan som börjar på den bokstaven. Undvika namn, såsom namn på personer, namn på länder o.s.v. samt ord med olika ändelser, såsom lång, längre, längst. Bokstaven är ” D, S, P, D , S, D, D.**  Ta tid i 30 sekunder med ett stoppur och stoppa därefter patienten.  POÄNG_______ |  |
|  | |
|  | |
|  | |
|  | |
|  | |
|  | |
|  | |
|  | |
|  | |
|  | |
|  | |

**ADL- faktorer**

A1. Kan du stå utan hjälp?

1. Inte alls
2. Med svårighet
3. Med lätthet

A2. Kan du gå utan hjälp?

1. Inte alls
2. Med svårighet
3. Med lätthet

A3. Kan du äta och dricka utan hjälp?

1. Inte alls
2. Med svårighet
3. Med lätthet

A4. Kan du klä på dig utan hjälp?

1. Inte alls
2. Med svårighet
3. Med lätthet

**VI.	Patients upplevelse av operationen/narkosförloppet**

O1. Jag kommer att visa dig ett antal olika ansikten och vill att du anger vilket ansikte, siffra eller beskrivning som bäst beskriver i vilken omfattning din operation negativt har påverkat din förmåga att arbeta, jämfört med innan operationen.

1. Ingen påverkan
2. Minimal påverkan
3. Måttlig påverkan
4. Stor påverkan
5. Uttalad påverkan, *kan inte arbeta*

O2. Jag kommer att visa dig ett antal olika ansikten och vill att du anger vilket ansikte, siffra eller beskrivning som bäst beskriver i vilken omfattning din operation negativt har påverkat din förmåga att utföra dina dagliga aktiviteter, jämfört med innan operationen.

1. Ingen påverkan
2. Minimal påverkan
3. Måttlig påverkan
4. Stor påverkan
5. Uttalad påverkan, *mitt dagliga liv är helt förändrat*

O3. Jag kommer att visa dig ett antal olika ansikten och vill att du anger vilket ansikte, siffra eller beskrivning som bäst beskriver i vilken omfattning din operation negativt har påverkat din förmåga att tänka klart, jämfört med innan operationen.

1. Ingen påverkan
2. Minimal påverkan
3. Måttlig påverkan
4. Stor påverkan
5. Uttalad påverkan, *mycket uttalad påverkan*

O4. Jag kommer att visa dig ett antal olika ansikten och vill att du anger vilket ansikte, siffra eller beskrivning som bäst beskriver hur nöjd du är med den narkos som du har fått.

1. Helt nöjd
2. Nöjd
3. Måttligt nöjd
4. Ganska nöjd
5. Inte alls nöjd
