# Supplementary material for: Evaluation of the Postoperative Quality of Recovery Scale test and re-test in Swedish among healthy volunteers
Source: F1000Res. 2016 Oct 21;5:2549. [Version 1] doi: 10.12688/f1000research.9740.1 (PMC5310376; doi:10.12688/f1000research.9740.1)
Supplement: Peer review history

# Peer review: Referee response for version 1

**DOI:** 10.5256/f1000research.10501.r20172

**Published:** 2017-02-13

**Authors:** Stanton Newman

**Affiliations:** 1 Centre for Health Services Research, School of Health Sciences, City University London, London, UK

This is a well conducted study examining changes over time in a newly translated Swedish version of the PostopQRS Scale. Sixty one students were assessed twice on the scale with a 48 hour gap between assessments. The physiological aspects of the PostopQRS Scale were not used.  On the first occasion participants were assessed face to face and the second via telephone. The PostopQRS Scale has been validated for telephone use in its English version.

 The authors report some learning on the tests of word recall and word generation. It is not uncommon to find some learning taking place on cognitive tests especially between the first and second occasions. This has been attributed in part to the fact that on the first occasion the tests are new and the participant does not know what to expect. On the second occasion the experience of having done the test enables some participants to adjust their strategy and obtain an improved score. The use of parallel forms is widely used to reduce learning of the actual items on any of the tests and the authors note that they may have increased the learning observed on these two tests by not using a parallel set of words on the word recall task and not counterbalancing the letter on Word Generation task.

 The authors note then baseline levels of pain, anxiety and depression along with highly variable cognitive performance all of which attest to the importance of having baseline scores before surgery in order to account for individual differences.  Overall the Swedish version of the PostopQRS appears to perform well.

I have read this submission. I believe that I have an appropriate level of expertise to confirm that it is of an acceptable scientific standard.

# Peer review: Referee response for version 1

**DOI:** 10.5256/f1000research.10501.r17147

**Published:** 2016-10-26

**Authors:** Colin F. Royse

**Affiliations:** 1 Department of Surgery, University of Melbourne, Melbourne, Vic, Australia

The authors have translated the PostopQRS into Swedish and conducted a validation study in normal volunteers. The study is well conducted and supports the translated version. Further, they have shown a small learning effect as well as considerable variation in baseline performance of the Scale, which emphasizes the need to always compare postoperative recovery against the individual's own baseline scores.

 The authors should mention in their discussion that their cognitive scores were very similar to the cognitive scores reported by Royse *et al* in their study on volunteers, indicating that the value of the scores in Swedish should be comparable to English version publications. ^1^

I have read this submission. I believe that I have an appropriate level of expertise to confirm that it is of an acceptable scientific standard.

## References

[1] RoyseCFNewmanSWilliamsZWilkinsonDJ: A human volunteer study to identify variability in performance in the cognitive domain of the postoperative quality of recovery scale. *Anesthesiology*.2013;119(3) : 10.1097/ALN.0b013e318299f72b 576-81 10.1097/ALN.0b013e318299f72b 23695174
